# Fracture Risk Associated With Dimethyl Fumarate Treatment in Multiple Sclerosis Patients: Population Heterogeneity and Temporal Patterns

**DOI:** 10.1111/cns.70612

**Published:** 2025-09-11

**Authors:** Dongdong He, Jingkai Di, Peirui Jiang, Lujia Liu, Feida Wang, Chuan Xiang

**Affiliations:** ^1^ Department of Orthopedics Second Hospital of Shanxi Medical University Taiyuan China; ^2^ Shanxi Provincial Key Laboratory of Bone and Soft Tissue Injury Repair Taiyuan China; ^3^ School of Clinical Medicine Shanxi Medical University Shanxi China; ^4^ School of Stomatology Shanxi Medical University Taiyuan China

**Keywords:** demographic disparities, dimethyl fumarate, fracture, multiple sclerosis, pharmacovigilance

## Abstract

**Objective:**

Dimethyl fumarate (DMF) is currently recommended as a first‐line therapy for multiple sclerosis (MS). Investigating its adverse events (AEs) holds significant clinical importance. This study aimed to explore the association between DMF and fracture‐related AEs.

**Methods:**

Data from the FDA Adverse Event Reporting System (FAERS) database (Q1 2004 to Q3 2024) were analyzed using disproportionality analysis. Reporting Odds Ratio (ROR), Proportional Reporting Ratio (PRR), Information Component (IC), and Empirical Bayes Geometric Mean (EBGM) were employed to identify and characterize AEs. Age‐ and gender‐specific subgroup analyses were conducted to evaluate AE patterns. Additionally, Weibull distribution analysis was performed to assess the temporal relationship of AE onset.

**Results:**

Overall, spinal fusion fracture (ROR = 13.13, 95% CI: 1.73–99.86) and coccyx fracture (ROR = 3.05, 95% CI: 1.92–4.86) exhibited the strongest signals. Gender and age heterogeneity were observed in DMF‐associated fracture risks. Female patients showed the highest signals for spinal fusion bone fracture (ROR = 13.13) and coccyx fracture (ROR = 2.80), while males predominantly exhibited patella (ROR = 4.94) and scapula fractures (ROR = 3.68). Age stratification revealed elevated risks of forearm fracture (ROR = 10.29, 95% CI: 4.24–24.96) and ankle fracture (ROR = 5.63, 95% CI: 4.32–7.35) in elderly patients, whereas younger populations displayed diverse multi‐site fractures. Time‐to‐onset (TTO) analysis indicated that 63.62% of fractures occurred within the first 2 years of the treatment cycle, with a median onset time of 506 days. Weibull distribution modeling indicated that DMF's failure pattern aligned with an early failure‐type curve.

**Conclusion:**

DMF use is associated with an increased risk of fracture‐related AEs, with demographic variations in susceptibility. This study provides critical insights into the safety profile of DMF in MS management, underscoring the need for vigilance in high‐risk populations.

## Graphical abstract

Schematic diagram of adverse events associated with fracture induced by dimethyl fumarate. This study further revealed the mechanism and safety of dimethyl fumarate through the discussion of real‐world data. The results showed that fracture‐related adverse events were common after the use of dimethyl fumarate. In addition, the warning signs of fracture were stronger in women and older adults after taking dimethyl fumarate.

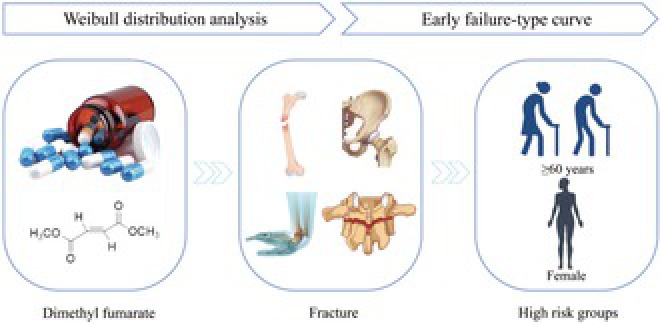

## 1 Introduction

Multiple sclerosis (MS) is a chronic inflammatory and demyelinating disease of the central nervous system (CNS), characterized by disseminated lesions across critical regions such as the brain, spinal cord, and optic nerves. These pathological changes lead to heterogeneous neurological dysfunction and progressive disability [1]. MS has emerged as a significant global public health challenge, with rising incidence, mortality, and disability‐adjusted life years (DALYs). The estimated global prevalence of MS has increased from 2.1 million cases in 2008 to 3.2 million in 2024 [2].

Furthermore, MS imposes substantial economic and psychosocial burdens due to its high treatment costs, including long‐term pharmacotherapy, rehabilitation, and regular medical surveillance. A study investigating the disease burden of cognitive dysfunction in MS patients reported lifetime direct medical costs as high as USD 58,200 per patient [3]. As the disease progresses, patients often experience loss of autonomy and face escalating risks of severe disability. Consequently, optimizing therapeutic strategies for MS remains a critical and urgent priority in clinical practice and research.

Current clinical management of MS centers on disease‐modifying therapies (DMTs), with pharmacological interventions constituting the cornerstone of MS treatment [4]. Dimethyl fumarate (DMF), the third oral disease‐modifying agent following fingolimod and teriflunomide, has emerged as a first‐line therapy for relapsing–remitting MS. Its therapeutic efficacy stems from Nrf2 pathway activation and subsequent induction of downstream antioxidant mechanisms, conferring both neuroprotective and immunomodulatory effects. Clinical evidence demonstrates that DMF achieves a 30% reduction in annualized relapse rate (ARR) compared to placebo, while offering advantages in oral administration convenience and a favorable cost‐effectiveness profile. These combined attributes have established DMF as a preferred therapeutic option in relapse management according to current treatment guidelines [5, 6].

Despite DMF's substantial therapeutic potential, as with any drug, it is inevitably accompanied by a range of possible adverse drug reactions (ADRs), and the long‐term safety of DMF still needs to be evaluated in depth. A clinical study of 2000 MS patients found that about 25% of patients experienced gastrointestinal discomfort and 15% developed rashes after using DMF, while liver dysfunction and blood system disorders occurred in 5% and 3% of patients, respectively. Additionally, in a long‐term efficacy study of DMF for the treatment of patients with MS, elevated liver transaminase levels were observed in approximately 8% of patients, and a small subset of patients (approximately 4%) exhibited decreased white blood cell (WBC) counts during the treatment period. In recent years, increased fracture risk has attracted attention as a potential drug safety issue. A cohort study showed that patients with MS treated with DMF for more than 2 years had a 40% increased incidence of fractures, mainly in the spine and hip. In addition, a study on the relationship between DMF and body metabolism has shown that long‐term use of DMF may lead to immune system disorders, which in turn lead to abnormal bone metabolism. Although there have been a few reports of DMF‐induced fractures, there is a lack of broad understanding of the extent of DMF's impact on the risk of fracture and how it varies across patient populations. Therefore, it is necessary to conduct further research on this issue.

The FDA Adverse Event Reporting System (FAERS) is a publicly accessible pharmacovigilance database containing voluntary adverse event (AE) reports submitted by consumers, manufacturers, healthcare professionals, and other stakeholders. This surveillance system is primarily designed to facilitate post‐marketing safety monitoring of biological products and therapeutic agents [7].

This study employs data mining analysis of FAERS‐reported AE cases to investigate fracture‐related adverse drug reactions associated with DMF therapy. Through systematic evaluation of real‐world safety signals, we aim to provide evidence‐based insights for clinical risk management and enhance medication safety in MS treatment.

## 2 Methods

### 2.1 Data Sources

**FIGURE 1:**
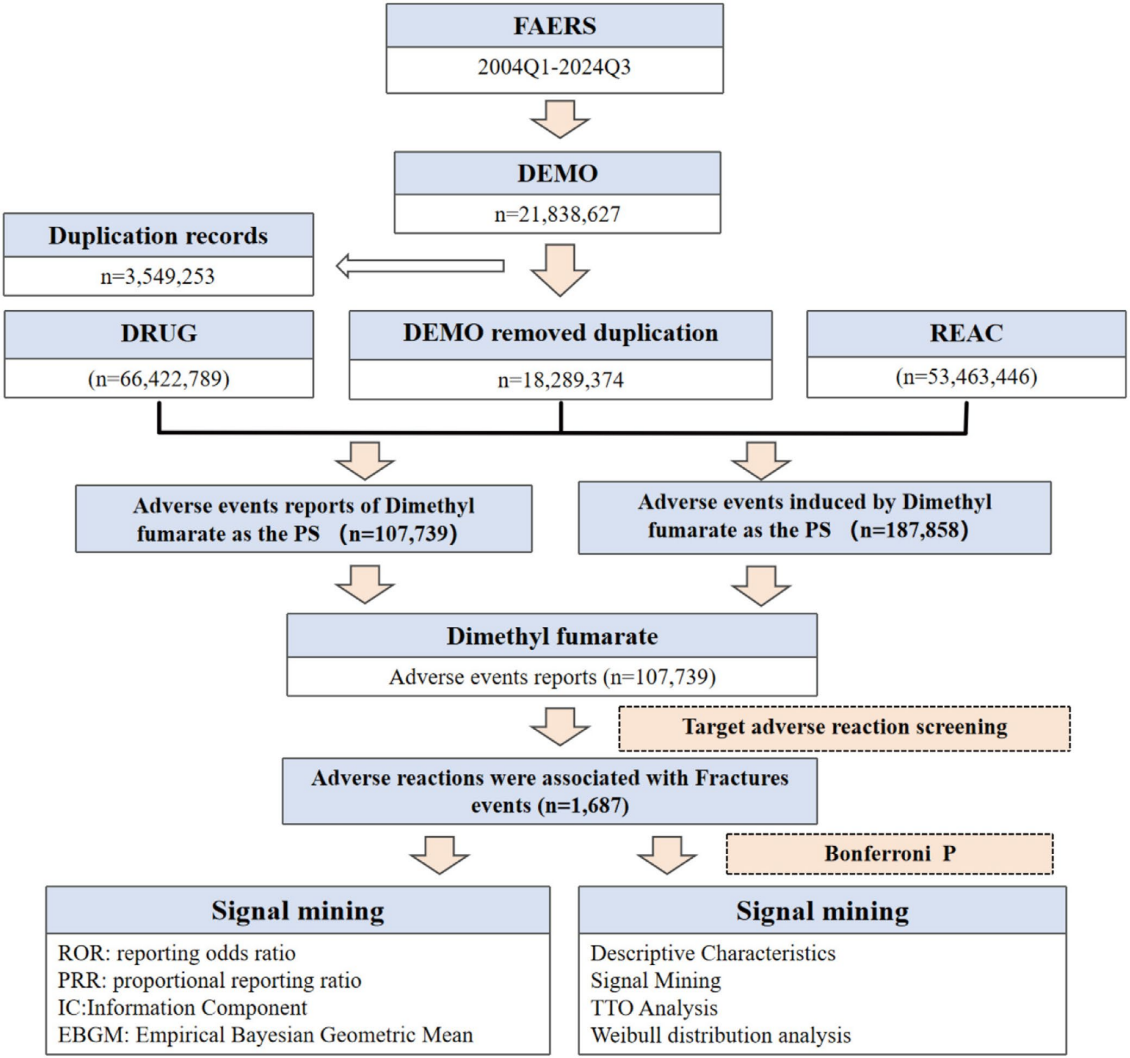
Schematic diagram of an adverse reaction to fracture caused by dimethyl fumarate.

Data from the open‐source FAERS spanning from the first quarter of 2004 to the third quarter of 2024 were adopted in this study. (Figure 1) The included content encompasses seven core subsets: demographic data (DEMO), drug exposures (DRUG), adverse event coding (REAC), outcomes (OUTC), report sources (RPSR), therapy duration windows (THER), and indications (INDI). These files were linked to relevant AEs through PRIMARYID, CASEID, and drug_seq to establish standardized association mechanisms. Additionally, this study utilized the International Safety Reporting Guidelines issued by the International Council for Harmonization of Technical Requirements for Pharmaceuticals for Human Use to adjust dynamic data.

### 2.2 Data Process

The Medical Dictionary for Regulatory Activities (MedDRA) Version 27.0 established a hierarchical medical terminology classification system encompassing Preferred Terms (PTs) for signs and symptoms, High‐Level Terms (HLT), High‐Level Group Terms (HLGT), and System Organ Classes (SOC), with multiple mapping associations constructed. This study primarily focused on the PT level. Among them, the MedDRA terminology classification criteria for adverse reactions categorize drugs into four classes: PS (Primary Suspect), SS (Secondary Suspect), C (Concomitant), and I (Interaction) [8, 9]. To ensure high precision in this study, we specifically concentrated on records marked with the drug role code “PS” (Primary Suspect) in the DRUG file. Simultaneously, comprehensive retrieval of dimethyl fumarate's generic and brand names was conducted through MeSH (Medical Subject Headings) descriptors to ensure data comprehensiveness for DMF. Notably, the raw data underwent rigorous cleaning procedures, whereby duplicate entries, records with missing critical fields, and non‐Primary Suspect (PS) drug records were excluded, thereby minimizing data bias and enhancing the depth and quality of data mining.

### 2.3 Time‐To‐Onset Analysis

Time‐to‐onset (TTO) is calculated as the duration from the patient's first prescription intake to the emergence of AEs associated with changes in anthropometric measurements. TTO data evaluation primarily relies on median values, mean values, and Weibull shape parameter (WSP) testing. Fluctuations in adverse event incidence following treatment initiation are influenced by the drug's mechanism of action and typically exhibit temporal variations with prolonged therapeutic duration. Considering the impact of pharmacological mechanisms on adverse event rates, WSP testing was applied to TTO statistical analyses to delineate temporal risk trends of increasing or decreasing adverse event probabilities. The shape parameter *β* of the Weibull distribution determines the configuration of the distribution function. Specifically, when *β* < 1 with its 95% confidence interval (CI) also < 1, AE incidence is considered to decrease over time, demonstrating characteristics of an early failure‐type curve. When *β* equals or approximates 1 with its 95% CI encompassing 1, AE incidence occurs randomly over time, manifesting as a random failure‐type curve. When *β* > 1 with its 95% CI excluding 1, AE incidence is interpreted as increasing over time, exhibiting a wear‐out failure‐type curve [10]. This pattern aligns with wear‐and‐tear failure mechanisms and proves particularly significant when investigating dimethyl fumarate's association with fractures.

### 2.4 Statistical Analysis

Disproportionality analysis as a signal detection methodology was employed for pharmacovigilance assessment in this study. Specifically, for the definition and identification of AEs, this research comprehensively utilized four methods: the reporting odds ratio (ROR), proportional reporting ratio (PRR), information component (IC), and empirical Bayesian geometric mean (EBGM). ROR controlled confounding biases such as age, gender, and combined medication through the unconditional logistic regression model, and its significance threshold was set as the lower limit of the 95% confidence interval (ROR05) > 1. PRR uses the Yates‐corrected chi‐square test to identify the temporal aggregation of high‐frequency events. The threshold setting needs to simultaneously satisfy PRR ≥ 2, *χ* ^2^ ≥ 4, and the number of basic reports *N* ≥ 3 [11]. For the zero‐expansion distribution feature, the IC is probabilistically inferred through the Bayesian recursive algorithm, and IC_025_ > 0 is established as the statistical significance criterion [12]. The EBGM adopts the threshold criterion of EBGM_05_ > 2 and corrects the small sample bias by contracting the estimator [13]. The above four methods are currently recognized as the commonly used methods for pharmacovigilance. The larger the signal value, the higher the intensity of the adverse drug event [14]. Among them, ROR has been verified by the guidelines of the European Medicines Agency (EMA) as suitable for low‐frequency but clinically significant associated detection. Therefore, we mainly use this method to evaluate the signal strength [15]. The calculation formulas for ROR, PRR, IC, and EBGM, along with signal generation criteria and signal strength determination thresholds, are presented in Table S1. To comprehensively screen potential drug safety risks, this study considered any report meeting at least one criterion across these four methodologies and reaching the positive signal threshold as a positive adverse event report, in accordance with established pharmacovigilance information guidelines. Building upon standardized preliminary data preparation procedures, we focused on mining baseline data and subgroup data to precisely identify potential values within specific population datasets. In this study, R (version 4.4.2) and RStudio were utilized for specific data analyses.

## 3 Results

### 3.1 Descriptive Analysis

During the test period from the first quarter of 2004 to the third quarter of 2024, the FAERS database documented 107,739 DMF adverse event reports, including 1687 fracture events (1.57%) (Table 1). The majority of AEs were reported by consumers (*n* = 82,536, 76.6%), followed primarily by healthcare professionals (*n* = 3758, 3.5%). In adverse event reports associated with DMF, gender data were fully recorded for 101,118 patients. Among these, female patients constituted a higher proportion (*n* = 79,917, 74.2%) compared to male patients (*n* = 21,201, 19.7%). Notably, for fracture events specifically, the number of female patients (*n* = 1399) was approximately five times higher than that of male patients (*n* = 279). Furthermore, in the population distribution of fracture‐related AEs, younger patients accounted for 52.9% (*n* = 892) while older adults comprised 14.5% (*n* = 244).

**TABLE 1:** Demographics of adverse events in fractures following dimethyl fumarate use.

Characteristic	Total	SMQ‐fractures
Number of events	107,739	1687
Gender, *n*%		
Female	79,917 (74.2%)	1399 (82.9%)
Male	21,201 (19.7%)	279 (16.5%)
Unknown	6621 (6.1%)	9 (0.5%)
Age (year), *n*%		
< 18	271 (0.3%)	0 (0%)
18–64.9	48,124 (44.7%)	892 (52.9%)
65–85	5516 (5.1%)	243 (14.4%)
> 85	13 (0.0%)	1 (0.1%)
Unknown	53,815 (49.9%)	551 (32.7%)
OCCP_COD, *n*%		
CN	82,536 (76.6%)	1090 (64.6%)
HP	3758 (3.5%)	99 (5.9%)
LW	8 (0.0%)	1 (0.1%)
MD	11,883 (11.0%)	358 (21.2%)
OT	5291 (4.9%)	74 (4.4%)
PH	3179 (3.0%)	28 (1.7%)
RN	67 (0.1%)	0 (0%)
Unknown	1017 (0.9%)	37 (2.2%)

Abbreviations: CH, consumer; CN, consumer; HP, health professional; LW, Lawyer; MD, Physician; *N*, number of adverse event reported; OT, other health‐professional; PH, Pharmacist; RN, registered nurse.

### 3.2 Signal Mining

#### 3.2.1 Accumulation of Adverse Event Signals Across and Organ Classes (SOC) Analyze

**FIGURE 2:**
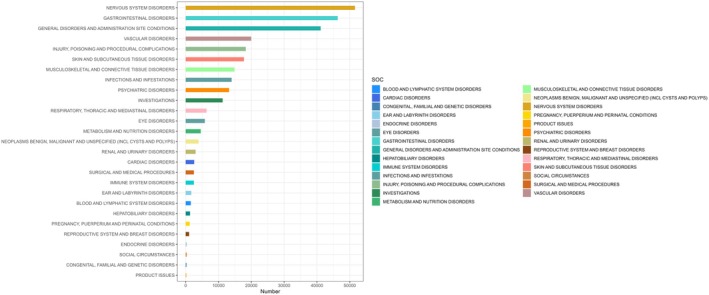
Bar Chart showing the number of AE cases reported for each SOC of Dimethyl fumarate. At the System Organ Class (SOC) level, a total of 27 organ systems were involved in dimethyl fumarate‐associated adverse events. CI, confidence interval; FAERS, Food and Drug Administration Adverse Event Reporting System; ROR, reporting odds ratio; SOC, systemic organ classification.

At the System Organ Class (SOC) level, a total of 27 organ systems were involved in DMF‐associated AEs (Figure 2). Among these, Nervous system disorders (*n* = 51,585) and Gastrointestinal disorders (*n* = 46,331) represented the most frequently accumulated systems in the population. Product issues accounted for the smallest number within SOCs (*n* = 193). Regarding adverse event signal strength, Vascular disorders demonstrated the strongest positive signal (ROR = 3.39), followed by Nervous system disorders (ROR = 2.35), with product issues exhibiting the weakest positive signal (ROR = 0.04).

#### 3.2.2 Analysis of Adverse Events at Preferred Term (PT) Signal Level

**FIGURE 3:**
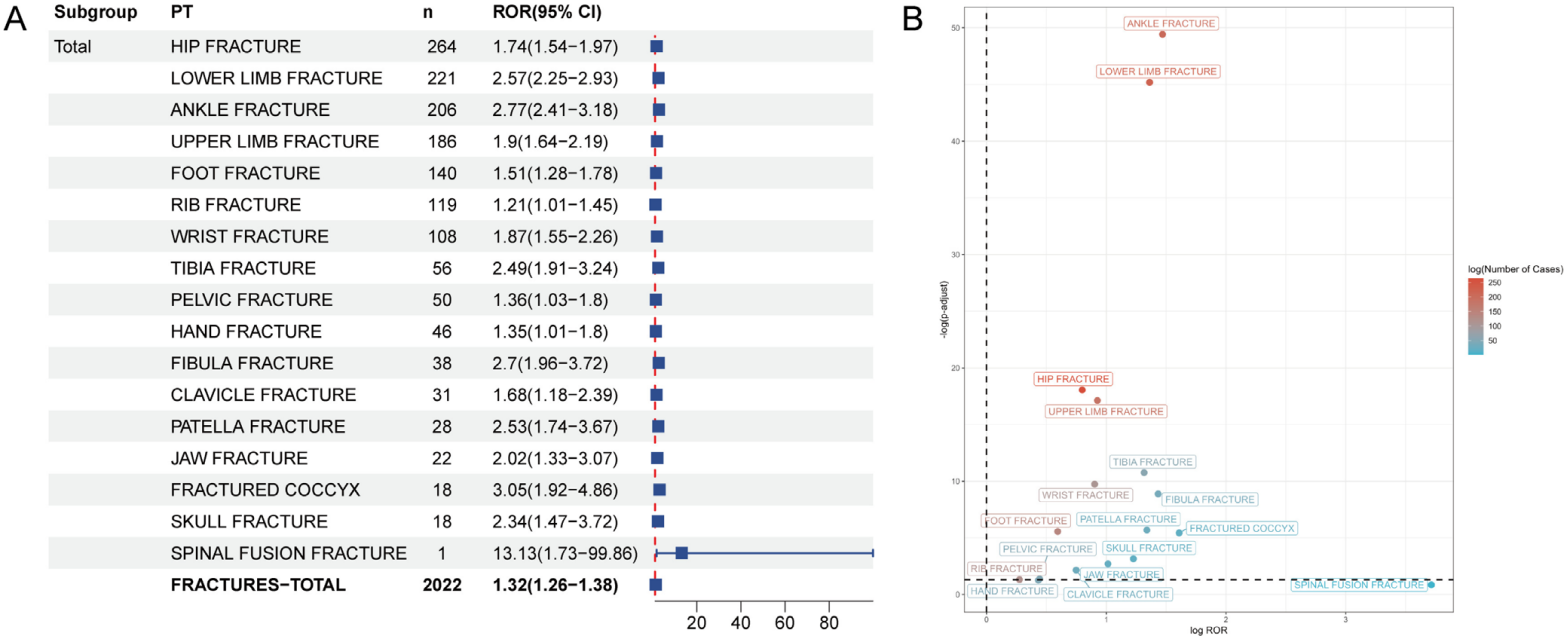
Overall analysis of adverse fracture reactions caused by Dimethyl fumarate. (A) The RORs and 95% CIs of 18 positive PT signals related to fractures were presented in the forest maps. (B) The Scatter Plot shows the distribution of 18 positive PT signals related to fractures. CI, confidence interval; PT, Preferred Terms; ROR, reporting odds ratio.

At the PT level, we evaluated a total of 49 distinct Preferred Terms (PTs), identifying 18 positive PT signals associated with fractures (Figure 3). Among these, spinal fusion fracture demonstrated the strongest signal strength (ROR = 13.13, 95% CI: 1.73–99.86). Additionally, a coccyx fracture was also identified with a high positive signal (ROR = 3.05, 95% CI: 1.92–4.86). Rib fracture exhibited the weakest signal strength among the 18 positive PTs (ROR = 1.21, 95% CI: 1.01–1.45).

Furthermore, we further calculated the signal association between DMF and overall fracture events. The results demonstrated that DMF maintained a positive signal in overall fracture‐related AEs (ROR: 1.32, 95% CI: 1.26–1.38).

#### 3.2.3 Subgroup Stratification Analysis

**FIGURE 4:**
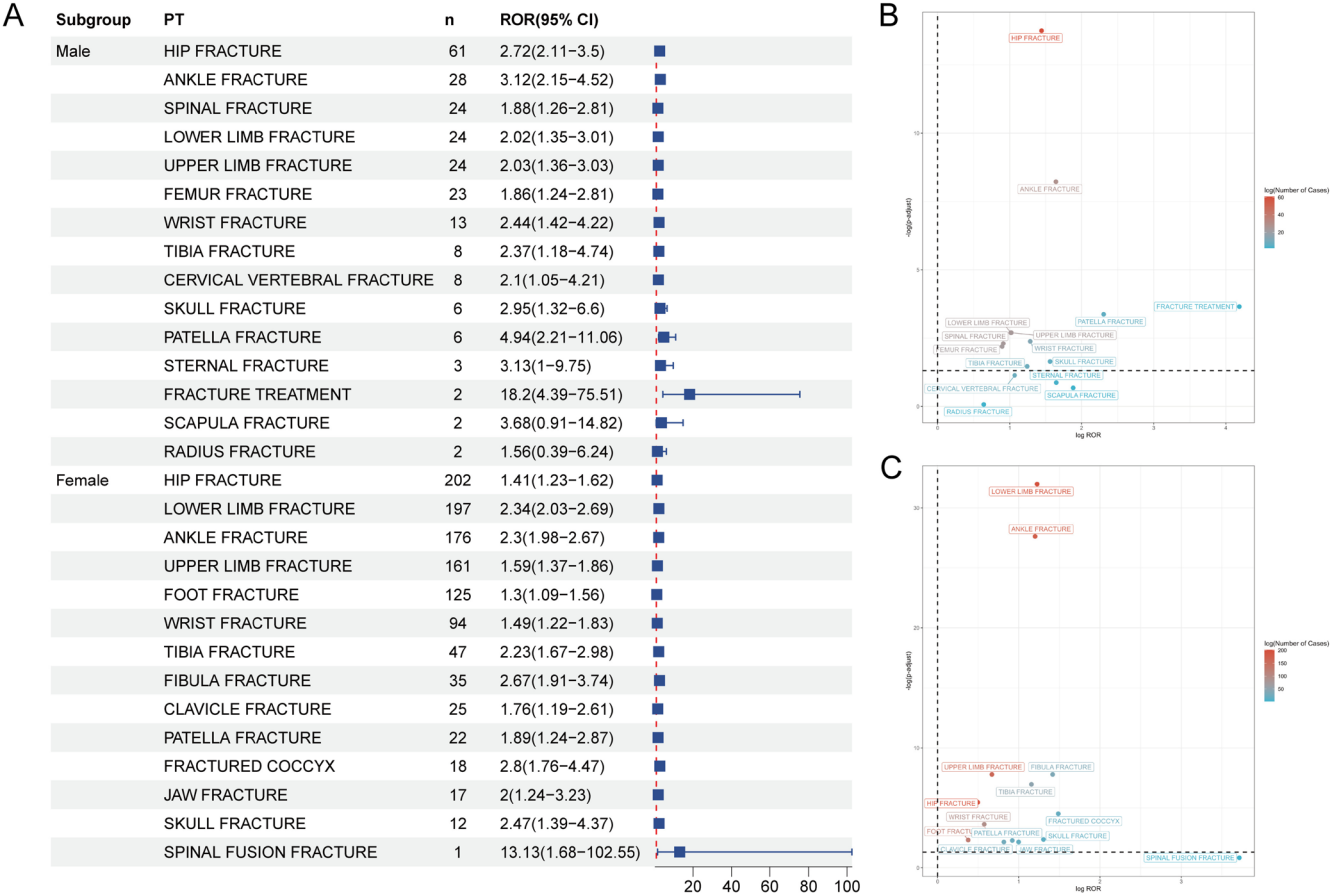
Analysis of sex‐differentiated risk signals in dimethyl fumarate. (A) The forest plot shows the RORs and 95% CIs of positive PT related to gender. (B) The scatter plot shows the risk signal of dimethyl fumarate‐induced fractures in the male subgroup. (C) The scatter plot shows the risk signal of dimethyl fumarate causing fractures in the female subgroup. CI, confidence interval; PT, Preferred terms; ROR, reporting odds ratio.

**FIGURE 5:**
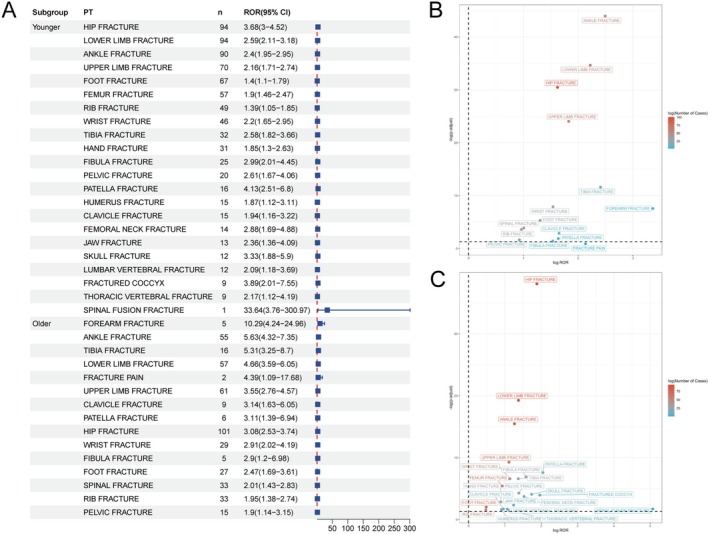
Analysis of age‐differentiated risk signals in dimethyl fumarate. (A) The forest plot shows the RORs and 95% CIs of all age‐related positive adverse events. (B) The scatter plot shows the PT types related to fractures in the subgroup of ≥ 60 years old. (C) The scatter plot shows the PT types related to fractures in the subgroup of < 60 years old. CI, confidence interval; PT, Preferred Terms; ROR, reporting odds ratio.

Further, we conducted stratified analyses across populations of different genders and age groups. Specifically, within the male subgroup, the highest AE signal strength was observed for patella fracture (ROR = 4.94, 95% CI: 2.21–11.06), followed by scapula fracture (ROR = 3.68, 95% CI: 0.91–14.82). In the female subgroup, the two AEs with the strongest AE signal strengths were spinal fusion fracture (ROR = 13.13, 95% CI: 1.68–102.55) and coccyx fracture (ROR = 2.8, 95% CI: 1.76–4.47). Spinal fusion fracture, coccyx fracture, fibula fracture, jaw fracture, clavicle fracture, foot fracture, hand fracture, and vertebral compression fracture were AEs unique to females. Scapula fracture, sternum fracture, cervical vertebral fracture, upper limb fracture, spinal fracture, and femur fracture were AEs exclusive to males (Figure 4). Additionally, age subgroup analyses revealed distinct patterns (Figure 5). In the population aged < 60 years, DMF‐associated fracture‐related AEs predominantly manifested as spinal fusion fracture (ROR = 33.64, 95% CI: 3.76–300.97) and patella fracture (ROR = 4.13, 95% CI: 2.51–6.8). Conversely, in the population aged ≥ 60 years, AEs were primarily concentrated in forearm fracture (ROR = 10.29, 95% CI: 4.24–24.96) and ankle fracture (ROR = 5.63, 95% CI: 4.32–7.35). Similarly, age‐specific fracture AEs were identified: spinal fusion fracture, coccyx fracture, skull fracture, femoral neck fracture, jaw fracture, thoracic vertebral fracture, lumbar vertebral fracture, femur fracture, humerus fracture, and hand fracture were unique to the < 60‐year subgroup. In the ≥ 60‐year subgroup, two specific AEs included forearm fracture and spinal fracture (Table S2).

### 3.3 Time‐To‐Onset Analysis

**FIGURE 6:**
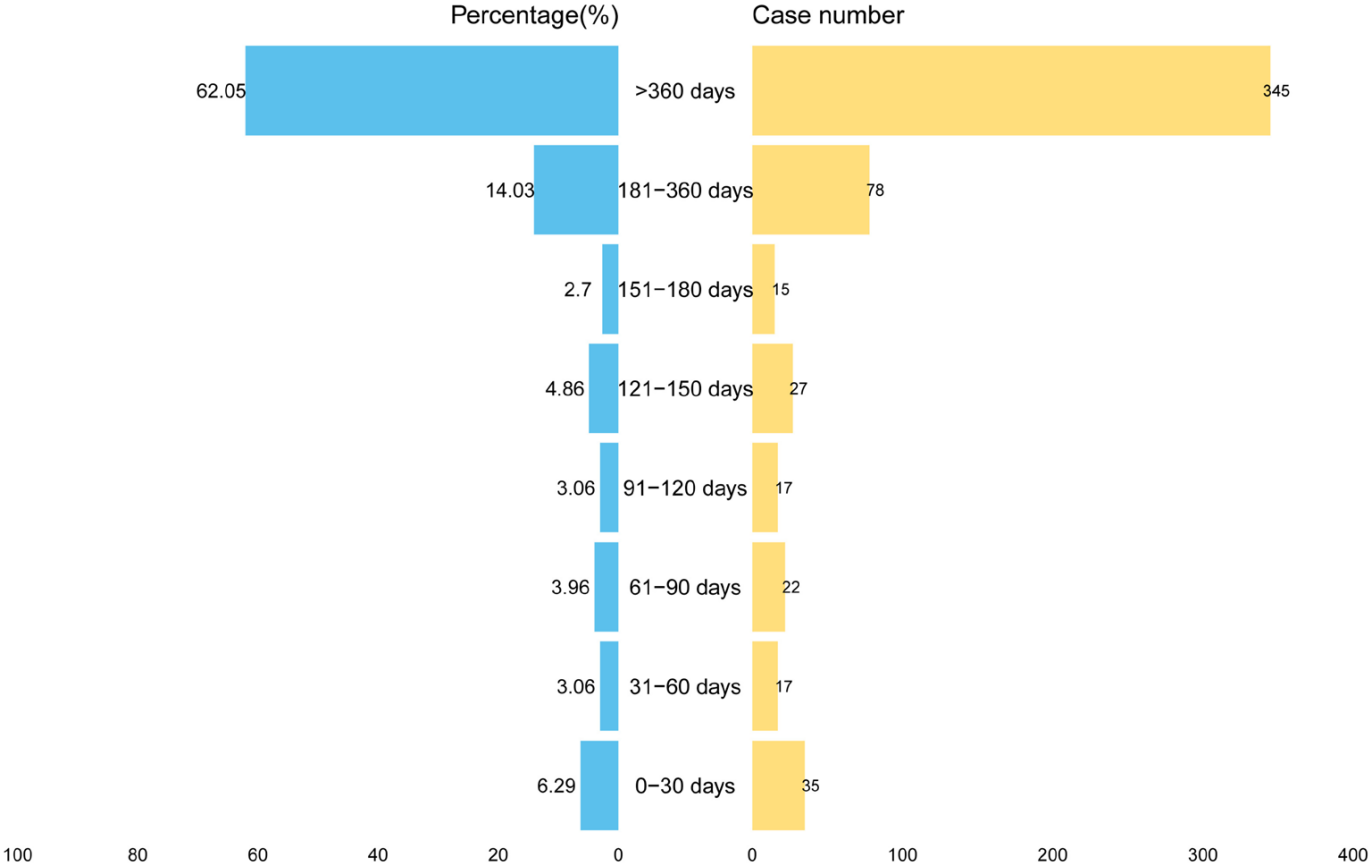
The butterfly plot shows the proportion and distribution of AE in different time intervals.

In our study, approximately 556 AE reports documented the time‐to‐onset (TTO) of adverse reactions, with a mean TTO of 801.79 ± 642.69 days and a median TTO of 506 days (Table 2 and Figure 6). About 72.4% of fracture events occurred within the first 3 years of treatment. Weibull distribution analysis revealed that the shape parameter *β* for the survival time of DMF‐associated fracture events was less than 1, with the 95% confidence interval (CI) upper limit also < 1, indicating that the failure‐type characteristics of DMF approximate an early failure‐type curve, i.e., the fracture AE incidence rate can be considered to decrease over time.

**TABLE 2:** Weibull shape parameter test for dimethyl fumarate.

Drug	Time‐to‐onset (days)			Weibull distribution		
	*N*	Median (IQR)	Min–Max	Scale parameter: *α* (95% CI)	Shape parameter: *β* (95% CI)	Failure type
Dimethyl fumarate	556	506 (188.75–1230)	2–3757	0.92 (0.86–0.99)	774.20 (700.99–847.42)	Early failure

## 4 Discussion

As DMF serves as a first‐line immunomodulatory agent for MS, investigating its adverse drug reactions (ADRs) holds critical importance. This study represents the first FAERS database‐driven demonstration that DMF treatment induces fracture‐related AEs, with signal strength demonstrating sex and age‐related heterogeneity. These findings underscore the necessity for fracture risk monitoring during MS treatment with this agent, while providing novel perspectives for understanding DMF's safety profile.

Our study suggests that DMF induces multiple fracture‐related AEs in the general population. The AEs with the strongest signal strengths were spinal fusion fracture and coccyx fracture. Among these, several potential mechanisms have been elucidated. Specifically, DMF, as an activator of the Nrf2 antioxidant pathway, can damage the energy metabolism homeostasis of osteocytes through long‐term exposure by interfering with mitochondrial function, ultimately inducing osteocyte apoptosis and weakening bone remodeling ability [16]. Furthermore, DMF is metabolized into the active intermediate monomethyl fumaric acid (MMF) in vivo, which can inhibit the NF‐κB and Wnt/β‐catenin signaling pathways [17]. This further significantly downregulated the expressions of the key transcription factors Runx2 and Osterix for osteoblast differentiation, resulting in a significant reduction in the rate of bone formation and exacerbating the imbalance of osteoclast‐mediated bone remodeling [18]. Concurrently, DMF has been found to suppress hypoxia‐inducible factor‐1α (HIF‐1α) expression and function in osteoblasts—this key molecule plays essential roles in fracture healing processes, with its inhibition directly impeding fracture repair [19]. Furthermore, DMF induces CYP24A1 enzyme overexpression, accelerating vitamin D metabolism toward the inactive form 24,25(OH)_2_D_3_. This metabolic shift not only disrupts normal vitamin D homeostasis but also reduces intestinal calcium absorption efficiency, thereby further compromising bone strength [20].

Existing evidence indicates that patients with MS inherently exhibit elevated fracture risk, primarily attributed to the complex effects of MS on the skeletal system during disease progression [21]. A clinical evidence shows that a 54‐year‐old female patient with relapses of multiple sclerosis (RRMS), after receiving continuous treatment with DMF for 30 months, complained of recurrent non‐traumatic lower limb bone pain and was diagnosed with bone marrow edema syndrome (BMES) by imaging examination [22]. While BMES may indirectly increase the risk of fractures through decreased bone strength, stress concentration, and osteoporosis. Specifically, elevated levels of proinflammatory cytokines such as IL‐6 and TNF‐α within the chronic neuroinflammatory microenvironment activate the RANKL/OPG signaling axis, promoting osteoclast differentiation and inhibiting osteoprotegerin synthesis [23]. This study reveals that the use of DMF also contributes to the occurrence of multiple fracture‐related AEs. Therefore, it is recommended to implement fracture risk assessment and preventive measures for MS patients receiving DMF treatment in clinical practice to mitigate fracture risk. Furthermore, combination therapy with targeted anti‐resorptive agents is proposed, as this integrated approach may synergistically inhibit neuroinflammatory bone loss and drug‐induced metabolic disturbances, thereby optimizing therapeutic safety.

Regarding gender differences, DMF‐associated AEs demonstrated higher occurrence rates in female patients (74.2%) compared to males (19.7%), a finding consistent with previous studies on MS and fracture risk. Notably, female patients exhibited prominent risks for spinal fusion fractures and coccyx fractures, which may be associated with postmenopausal estrogen decline‐induced trabecular microstructure degeneration, particularly in vertebral bodies and other cancellous bone‐enriched regions [24]. The deficiency of estrogen can lead to an increase in IL‐7 to promote the activation of T cells. T cells induce the production of pro‐inflammatory molecules such as IL‐1, IL‐6, and TNF‐α, thereby resulting in the formation of osteoclasts [25, 26]. In addition, the accumulation of reactive oxygen species (ROS) under low estrogen conditions can further enhance T cell activation and osteoclast generation and accelerate the production of tumor necrosis factor (TNF) [26]. Meanwhile, a cross‐sectional study involving 2723 subjects confirmed that the incidence of vitamin D deficiency in women was higher than that in men, and the efficiency of intestinal calcium absorption decreased, making them more prone to calcium loss and a decline in bone strength [27]. The gender‐specific predisposition to coccyx fractures might relate to female pelvic anatomical configuration, with additional contributions from pregnancy‐induced ligamentous laxity potentially altering stress distribution patterns during falls, thereby increasing shear forces on the coccyx [28]. Patella fractures and scapula fractures emerged as male‐specific AEs, possibly linked to androgen‐regulated cortical bone remodeling abnormalities. Reduced testosterone levels in males may impair periosteal osteoblast differentiation, consequently diminishing the torsional resistance capacity of long bones [29]. Furthermore, elevated scapula fracture risk may reflect occupational exposure disparities, as males demonstrate higher engagement in high‐impact activities, predisposing them to direct traumatic injuries.

Furthermore, this study identified age‐related associations between DMF use and fracture occurrence, with older adults demonstrating higher susceptibility compared to younger patients. This is consistent with the current view. The possible mechanism is that with the increase of age, the loss of cancellous bone and cortical bone in the elderly is severe, resulting in their bone density being lower than that of young people and making them more prone to the imbalance between osteoblasts and osteoclasts [30]. Specifically, AEs in older adults predominantly manifested as forearm fractures and spinal fractures. This may be attributed to age‐related declines in vitamin D synthesis capacity, reduced serum calcium levels, and inadequate bone mineralization, collectively resulting in significant increases in forearm bone fragility [31]. The forearm, being a cancellous bone‐rich region subjected to substantial mechanical loading, becomes particularly vulnerable to fractures from low‐impact falls (e.g., hand impact during ground contact). In contrast, younger patients exhibited diverse fracture types across multiple anatomical sites, likely stemming from their higher engagement in vigorous physical activities and high‐risk sports, compounded by external traumatic injuries such as traffic accidents, leading to greater heterogeneity in fracture locations and patterns [31].

TTO analysis revealed that the majority of AEs (63.62%) occurred within the first 24 months following DMF initiation. Furthermore, WSP testing demonstrated that DMF's Weibull shape parameter *β* < 1 with its 95% CI < 1, consistent with characteristics of an early failure‐type curve, indicating a temporal decline in adverse event incidence. These findings align with previous studies showing reduced DMF‐associated adverse event occurrence over time. Consequently, heightened vigilance for AEs is warranted during the initial DMF treatment phase, particularly within the first 2 years, requiring prompt identification, early intervention, and implementation of appropriate supportive care to alleviate symptoms and prevent severe complications. Notably, Weibull analysis results suggest progressive attenuation of adverse event signal strength with prolonged treatment duration, thereby validating DMF's safety profile and therapeutic reliability in long‐term MS management.

This study possesses several notable strengths. First, we employed disproportionality analysis methodology, which enables effective identification of adverse reactions and potential risks not documented in drug prescribing information. Furthermore, through an in‐depth exploration of the mechanisms and influencing factors underlying these adverse reactions, this investigation further elucidates the drug's pharmacological mechanisms and safety concerns, thereby establishing a scientific foundation for developing safer and more effective therapeutic agents.

Nevertheless, certain limitations warrant consideration. First, the FAERS database primarily relies on spontaneous reporting, which may introduce reporting bias. Additionally, sampling bias may exist, particularly in regions with higher reporting frequencies, as not all AEs are comprehensively captured. Meanwhile, at present, there are relatively few relevant clinical studies that can fully prove the clear association between DMF and the occurrence of fractures, and extensive exploration of this issue is still needed. Importantly, this study did not quantify absolute risks nor infer definitive causal relationships, providing only statistically significant signal strength estimates. Moreover, due to potential confounding from patients' underlying comorbidities, prospective cohort studies remain necessary to quantify fracture risks and explore the feasibility of preventive strategies. Concurrently, continuous surveillance is required to assess the potential emergence of other uncharacterized AEs.

## 5 Conclusion

This study analyzed the temporal patterns and safety profile of DMF‐associated fracture‐related AEs using the FAERS database. DMF demonstrated significant sexual dimorphism, age‐related heterogeneity, and time‐dependent characteristics in fracture risk, with underlying mechanisms involving osteogenic suppression, oxidative damage, and calcium homeostasis dysregulation. Clinicians should maintain heightened vigilance for these AEs and develop personalized intervention strategies for high‐risk subgroups. Given the inherent biases of spontaneous reporting databases, further clinical investigations and longitudinal data remain imperative to validate these findings and comprehensively characterize DMF's long‐term safety profile.

## Author Contributions

**Dongdong He:** writing – original draft, methodology, conceptualization. **Jingkai Di:** writing – review and editing, validation. **Peirui Jiang:** writing – review and editing. **Lujia Liu:** writing – review and editing. **Feida Wang:** writing – review and editing. **Chuan Xiang:** writing – review and editing, methodology, funding acquisition, formal analysis, data curation, conceptualization.

## Ethics Statement

The authors have nothing to report.

## Conflicts of Interest

The authors declare no conflicts of interest.

## Supporting information


**Table S1:** Four major algorithms used for signal detection.

**Table S2:** PT levels signal strength of dimethyl fumarate adverse event reports and subgroup analysis based on age and gender. EBGM, empirical bayesian geometric mean; IC, information component; *p* value, Adjusted *p* value; PRR, proportional reporting ratio; ROR, report odds ratio.

## Data Availability

Data for the FAERS database were obtained and extracted from Q1 2004 to Q3 2024. The open‐source database used to conduct the study is available on the PMDA website (https://www.fda.gov).
