# Supplementary material for: Fracture Risk Associated With Dimethyl Fumarate Treatment in Multiple Sclerosis Patients: Population Heterogeneity and Temporal Patterns
Source: CNS Neurosci Ther. 2025 Sep 11;31(9):e70612. doi: 10.1111/cns.70612 (PMC12426412; doi:10.1111/cns.70612)
Supplement: Supplementary file 2 — Table S2: PT levels signal strength of dimethyl fumarate adverse event reports and subgroup analysis based on age and gender. EBGM, empirical bayesian geometric mean; IC, information component; p value, Adjusted p value; PRR, proportional reporting ratio; ROR, report odds ratio. [file CNS-31-e70612-s002.docx]

**Supplementary Material Table 2** PT levels signal strength of dimethyl fumarate adverse event reports and subgroup analysis based

on age and gender

| **Item** | **AEs** | **N** | **ROR(95%Cl)** | **PRR(χ²)** | **EBGM(EBGM05)** | **IC(IC025)** |
| --- | --- | --- | --- | --- | --- | --- |
| **Total** | HIP FRACTURE | 264 | 1.74 ( 1.54 - 1.97 ) | 1.74 ( 82.48 ) | 1.73 ( 1.57 ) | 0.79 ( 0.62 ) |
|  | LOWER LIMB FRACTURE | 221 | 2.57 ( 2.25 - 2.93 ) | 2.56 ( 208.13 ) | 2.54 ( 2.28 ) | 1.35 ( 1.15 ) |
|  | ANKLE FRACTURE | 206 | 2.77 ( 2.41 - 3.18 ) | 2.77 ( 229.12 ) | 2.74 ( 2.44 ) | 1.45 ( 1.25 ) |
|  | UPPER LIMB FRACTURE | 186 | 1.9 ( 1.64 - 2.19 ) | 1.89 ( 77.79 ) | 1.89 ( 1.67 ) | 0.91 ( 0.7 ) |
|  | FOOT FRACTURE | 140 | 1.51 ( 1.28 - 1.78 ) | 1.51 ( 23.7 ) | 1.5 ( 1.31 ) | 0.59 ( 0.34 ) |
|  | RIB FRACTURE | 119 | 1.21 ( 1.01 - 1.45 ) | 1.21 ( 4.32 ) | 1.21 ( 1.04 ) | 0.27 ( 0.01 ) |
|  | WRIST FRACTURE | 108 | 1.87 ( 1.55 - 2.26 ) | 1.87 ( 43.53 ) | 1.86 ( 1.59 ) | 0.9 ( 0.62 ) |
|  | TIBIA FRACTURE | 56 | 2.49 ( 1.91 - 3.24 ) | 2.49 ( 49.05 ) | 2.47 ( 1.98 ) | 1.3 ( 0.92 ) |
|  | PELVIC FRACTURE | 50 | 1.36 ( 1.03 - 1.8 ) | 1.36 ( 4.81 ) | 1.36 ( 1.08 ) | 0.44 ( 0.04 ) |
|  | HAND FRACTURE | 46 | 1.35 ( 1.01 - 1.8 ) | 1.35 ( 4.14 ) | 1.35 ( 1.06 ) | 0.43 ( 0.01 ) |
|  | FIBULA FRACTURE | 38 | 2.7 ( 1.96 - 3.72 ) | 2.7 ( 40.13 ) | 2.68 ( 2.05 ) | 1.42 ( 0.95 ) |
|  | CLAVICLE FRACTURE | 31 | 1.68 ( 1.18 - 2.39 ) | 1.68 ( 8.37 ) | 1.67 ( 1.24 ) | 0.74 ( 0.23 ) |
|  | PATELLA FRACTURE | 28 | 2.53 ( 1.74 - 3.67 ) | 2.53 ( 25.48 ) | 2.51 ( 1.83 ) | 1.33 ( 0.79 ) |
|  | JAW FRACTURE | 22 | 2.02 ( 1.33 - 3.07 ) | 2.02 ( 11.19 ) | 2.01 ( 1.41 ) | 1.01 ( 0.4 ) |
|  | FRACTURED COCCYX | 18 | 3.05 ( 1.92 - 4.86 ) | 3.05 ( 24.44 ) | 3.02 ( 2.04 ) | 1.59 ( 0.93 ) |
|  | SKULL FRACTURE | 18 | 2.34 ( 1.47 - 3.72 ) | 2.34 ( 13.57 ) | 2.32 ( 1.57 ) | 1.21 ( 0.55 ) |
|  | SPINAL FUSION FRACTURE | 1 | 13.13 ( 1.73 - 99.86 ) | 13.13 ( 10.46 ) | 12.32 ( 2.26 ) | 3.62 ( 1.46 ) |
|  | **FRACTURES−TOTAL** | 2022 | 1.32 ( 1.26 - 1.38 ) | 1.32 ( 152.81 ) | 1.31 ( 1.27 ) | 0.39 ( 0.33 ) |

| **Item** | **AEs** | **N** | **ROR(95%Cl)** | **PRR(χ²)** | **EBGM(EBGM05)** | **IC(IC025)** |
| --- | --- | --- | --- | --- | --- | --- |
| **Male** | HIP FRACTURE | 61 | 2.72 ( 2.11 - 3.5 ) | 2.72 ( 65.75 ) | 2.7 ( 2.19 ) | 1.44 ( 1.07 ) |
|  | ANKLE FRACTURE | 28 | 3.12 ( 2.15 - 4.52 ) | 3.11 ( 39.83 ) | 3.09 ( 2.27 ) | 1.63 ( 1.09 ) |
|  | SPINAL FRACTURE | 24 | 1.88 ( 1.26 - 2.81 ) | 1.88 ( 9.88 ) | 1.88 ( 1.34 ) | 0.91 ( 0.33 ) |
|  | LOWER LIMB FRACTURE | 24 | 2.02 ( 1.35 - 3.01 ) | 2.02 ( 12.23 ) | 2.01 ( 1.44 ) | 1.01 ( 0.43 ) |
|  | UPPER LIMB FRACTURE | 24 | 2.03 ( 1.36 - 3.03 ) | 2.03 ( 12.39 ) | 2.02 ( 1.44 ) | 1.01 ( 0.43 ) |
|  | FEMUR FRACTURE | 23 | 1.86 ( 1.24 - 2.81 ) | 1.86 ( 9.16 ) | 1.86 ( 1.32 ) | 0.89 ( 0.3 ) |
|  | WRIST FRACTURE | 13 | 2.44 ( 1.42 - 4.22 ) | 2.44 ( 10.99 ) | 2.43 ( 1.54 ) | 1.28 ( 0.51 ) |
|  | TIBIA FRACTURE | 8 | 2.37 ( 1.18 - 4.74 ) | 2.37 ( 6.27 ) | 2.36 ( 1.32 ) | 1.24 ( 0.27 ) |
|  | CERVICAL VERTEBRAL FRACTURE | 8 | 2.1 ( 1.05 - 4.21 ) | 2.1 ( 4.6 ) | 2.1 ( 1.17 ) | 1.07 ( 0.1 ) |
|  | SKULL FRACTURE | 6 | 2.95 ( 1.32 - 6.6 ) | 2.95 ( 7.68 ) | 2.94 ( 1.5 ) | 1.55 ( 0.46 ) |
|  | PATELLA FRACTURE | 6 | 4.94 ( 2.21 - 11.06 ) | 4.94 ( 18.58 ) | 4.88 ( 2.49 ) | 2.29 ( 1.19 ) |
|  | STERNAL FRACTURE | 3 | 3.13 ( 1 - 9.75 ) | 3.13 ( 4.3 ) | 3.11 ( 1.2 ) | 1.64 ( 0.18 ) |
|  | FRACTURE TREATMENT | 2 | 18.2 ( 4.39 - 75.51 ) | 18.2 ( 30.84 ) | 17.32 ( 5.26 ) | 4.11 ( 2.39 ) |
|  | SCAPULA FRACTURE | 2 | 3.68 ( 0.91 - 14.82 ) | 3.68 ( 3.86 ) | 3.65 ( 1.14 ) | 1.87 ( 0.19 ) |
|  | RADIUS FRACTURE | 2 | 1.56 ( 0.39 - 6.24 ) | 1.56 ( 0.39 ) | 1.55 ( 0.49 ) | 0.63 ( -1.04 ) |

| **Item** | **AEs** | **N** | **ROR(95%Cl)** | **PRR(χ²)** | **EBGM(EBGM05)** | **IC(IC025)** |
| --- | --- | --- | --- | --- | --- | --- |
| **Femal**e | HIP FRACTURE | 202 | 1.41 ( 1.23 - 1.62 ) | 1.41 ( 23.57 ) | 1.4 ( 1.25 ) | 0.49 ( 0.28 ) |
|  | LOWER LIMB FRACTURE | 197 | 2.34 ( 2.03 - 2.69 ) | 2.34 ( 148.42 ) | 2.32 ( 2.06 ) | 1.21 ( 1 ) |
|  | ANKLE FRACTURE | 176 | 2.3 ( 1.98 - 2.67 ) | 2.3 ( 127.14 ) | 2.28 ( 2.01 ) | 1.19 ( 0.97 ) |
|  | UPPER LIMB FRACTURE | 161 | 1.59 ( 1.37 - 1.86 ) | 1.59 ( 35.25 ) | 1.59 ( 1.39 ) | 0.67 ( 0.44 ) |
|  | FOOT FRACTURE | 125 | 1.3 ( 1.09 - 1.56 ) | 1.3 ( 8.81 ) | 1.3 ( 1.12 ) | 0.38 ( 0.12 ) |
|  | WRIST FRACTURE | 94 | 1.49 ( 1.22 - 1.83 ) | 1.49 ( 15.11 ) | 1.49 ( 1.25 ) | 0.57 ( 0.27 ) |
|  | TIBIA FRACTURE | 47 | 2.23 ( 1.67 - 2.98 ) | 2.23 ( 31.38 ) | 2.21 ( 1.74 ) | 1.14 ( 0.72 ) |
|  | FIBULA FRACTURE | 35 | 2.67 ( 1.91 - 3.74 ) | 2.67 ( 35.91 ) | 2.64 ( 1.99 ) | 1.4 ( 0.91 ) |
|  | CLAVICLE FRACTURE | 25 | 1.76 ( 1.19 - 2.61 ) | 1.76 ( 8.09 ) | 1.75 ( 1.26 ) | 0.81 ( 0.24 ) |
|  | PATELLA FRACTURE | 22 | 1.89 ( 1.24 - 2.87 ) | 1.89 ( 9.03 ) | 1.87 ( 1.32 ) | 0.91 ( 0.3 ) |
|  | FRACTURED COCCYX | 18 | 2.8 ( 1.76 - 4.47 ) | 2.8 ( 20.44 ) | 2.77 ( 1.87 ) | 1.47 ( 0.8 ) |
|  | JAW FRACTURE | 17 | 2 ( 1.24 - 3.23 ) | 2 ( 8.35 ) | 1.98 ( 1.33 ) | 0.99 ( 0.3 ) |
|  | SKULL FRACTURE | 12 | 2.47 ( 1.39 - 4.37 ) | 2.47 ( 10.3 ) | 2.44 ( 1.51 ) | 1.29 ( 0.48 ) |
|  | SPINAL FUSION FRACTURE | 1 | 13.13 ( 1.68 - 102.55 ) | 13.13 ( 10.18 ) | 12.02 ( 2.15 ) | 3.59 ( 1.38 ) |

| **Item** | **AEs** | **N** | **ROR(95%Cl)** | **PRR(χ²)** | **EBGM(EBGM05)** | **IC(IC025)** |
| --- | --- | --- | --- | --- | --- | --- |
| **≥60** | FOREARM FRACTURE | 5 | 10.29 ( 4.24 - 24.96 ) | 10.28 ( 40.98 ) | 10.08 ( 4.8 ) | 3.33 ( 2.14 ) |
|  | ANKLE FRACTURE | 55 | 5.63 ( 4.32 - 7.35 ) | 5.62 ( 206.58 ) | 5.57 ( 4.46 ) | 2.48 ( 2.09 ) |
|  | TIBIA FRACTURE | 16 | 5.31 ( 3.25 - 8.7 ) | 5.31 ( 55.38 ) | 5.26 ( 3.48 ) | 2.4 ( 1.69 ) |
|  | LOWER LIMB FRACTURE | 57 | 4.66 ( 3.59 - 6.05 ) | 4.65 ( 161.73 ) | 4.61 ( 3.71 ) | 2.21 ( 1.82 ) |
|  | FRACTURE PAIN | 2 | 4.39 ( 1.09 - 17.68 ) | 4.39 ( 5.19 ) | 4.36 ( 1.36 ) | 2.12 ( 0.45 ) |
|  | UPPER LIMB FRACTURE | 61 | 3.55 ( 2.76 - 4.57 ) | 3.55 ( 110.73 ) | 3.53 ( 2.86 ) | 1.82 ( 1.45 ) |
|  | CLAVICLE FRACTURE | 9 | 3.14 ( 1.63 - 6.05 ) | 3.14 ( 13.02 ) | 3.12 ( 1.8 ) | 1.64 ( 0.73 ) |
|  | PATELLA FRACTURE | 6 | 3.11 ( 1.39 - 6.94 ) | 3.11 ( 8.51 ) | 3.09 ( 1.58 ) | 1.63 ( 0.53 ) |
|  | HIP FRACTURE | 101 | 3.08 ( 2.53 - 3.74 ) | 3.07 ( 140.21 ) | 3.06 ( 2.59 ) | 1.61 ( 1.32 ) |
|  | WRIST FRACTURE | 29 | 2.91 ( 2.02 - 4.19 ) | 2.91 ( 36.01 ) | 2.89 ( 2.13 ) | 1.53 ( 1 ) |
|  | FIBULA FRACTURE | 5 | 2.9 ( 1.2 - 6.98 ) | 2.9 ( 6.17 ) | 2.88 ( 1.38 ) | 1.53 ( 0.35 ) |
|  | FOOT FRACTURE | 27 | 2.47 ( 1.69 - 3.61 ) | 2.47 ( 23.55 ) | 2.46 ( 1.8 ) | 1.3 ( 0.75 ) |
|  | SPINAL FRACTURE | 33 | 2.01 ( 1.43 - 2.83 ) | 2.01 ( 16.62 ) | 2 ( 1.5 ) | 1 ( 0.51 ) |
|  | RIB FRACTURE | 33 | 1.95 ( 1.38 - 2.74 ) | 1.95 ( 15.15 ) | 1.94 ( 1.46 ) | 0.96 ( 0.46 ) |
|  | PELVIC FRACTURE | 15 | 1.9 ( 1.14 - 3.15 ) | 1.9 ( 6.32 ) | 1.89 ( 1.24 ) | 0.92 ( 0.2 ) |

| **Item** | **AEs** | **N** | **ROR(95%Cl)** | **PRR(χ²)** | **EBGM(EBGM05)** | **IC(IC025)** |
| --- | --- | --- | --- | --- | --- | --- |
| **＜60** | HIP FRACTURE | 94 | 3.68 ( 3 - 4.52 ) | 3.68 ( 178.52 ) | 3.61 ( 3.04 ) | 1.85 ( 1.55 ) |
|  | LOWER LIMB FRACTURE | 94 | 2.59 ( 2.11 - 3.18 ) | 2.59 ( 90.14 ) | 2.56 ( 2.16 ) | 1.36 ( 1.06 ) |
|  | ANKLE FRACTURE | 90 | 2.4 ( 1.95 - 2.95 ) | 2.4 ( 71.91 ) | 2.37 ( 1.99 ) | 1.25 ( 0.94 ) |
|  | UPPER LIMB FRACTURE | 70 | 2.16 ( 1.71 - 2.74 ) | 2.16 ( 43.05 ) | 2.14 ( 1.76 ) | 1.1 ( 0.75 ) |
|  | FOOT FRACTURE | 67 | 1.4 ( 1.1 - 1.79 ) | 1.4 ( 7.72 ) | 1.4 ( 1.14 ) | 0.49 ( 0.13 ) |
|  | FEMUR FRACTURE | 57 | 1.9 ( 1.46 - 2.47 ) | 1.9 ( 23.98 ) | 1.89 ( 1.52 ) | 0.92 ( 0.54 ) |
|  | RIB FRACTURE | 49 | 1.39 ( 1.05 - 1.85 ) | 1.39 ( 5.39 ) | 1.39 ( 1.1 ) | 0.47 ( 0.06 ) |
|  | WRIST FRACTURE | 46 | 2.2 ( 1.65 - 2.95 ) | 2.2 ( 29.7 ) | 2.18 ( 1.71 ) | 1.13 ( 0.7 ) |
|  | TIBIA FRACTURE | 32 | 2.58 ( 1.82 - 3.66 ) | 2.58 ( 30.34 ) | 2.55 ( 1.9 ) | 1.35 ( 0.84 ) |
|  | HAND FRACTURE | 31 | 1.85 ( 1.3 - 2.63 ) | 1.85 ( 11.89 ) | 1.84 ( 1.36 ) | 0.88 ( 0.36 ) |
|  | FIBULA FRACTURE | 25 | 2.99 ( 2.01 - 4.45 ) | 2.99 ( 32.45 ) | 2.95 ( 2.12 ) | 1.56 ( 0.99 ) |
|  | PELVIC FRACTURE | 20 | 2.61 ( 1.67 - 4.06 ) | 2.61 ( 19.4 ) | 2.57 ( 1.78 ) | 1.36 ( 0.73 ) |
|  | PATELLA FRACTURE | 16 | 4.13 ( 2.51 - 6.8 ) | 4.13 ( 36.86 ) | 4.04 ( 2.66 ) | 2.01 ( 1.3 ) |
|  | HUMERUS FRACTURE | 15 | 1.87 ( 1.12 - 3.11 ) | 1.87 ( 5.99 ) | 1.86 ( 1.21 ) | 0.89 ( 0.17 ) |
|  | CLAVICLE FRACTURE | 15 | 1.94 ( 1.16 - 3.22 ) | 1.94 ( 6.7 ) | 1.92 ( 1.26 ) | 0.94 ( 0.22 ) |
|  | FEMORAL NECK FRACTURE | 14 | 2.88 ( 1.69 - 4.88 ) | 2.88 ( 16.77 ) | 2.84 ( 1.82 ) | 1.5 ( 0.75 ) |
|  | JAW FRACTURE | 13 | 2.36 ( 1.36 - 4.09 ) | 2.36 ( 10.02 ) | 2.34 ( 1.48 ) | 1.22 ( 0.45 ) |
|  | SKULL FRACTURE | 12 | 3.33 ( 1.88 - 5.9 ) | 3.33 ( 19.08 ) | 3.27 ( 2.03 ) | 1.71 ( 0.9 ) |
|  | LUMBAR VERTEBRAL FRACTURE | 12 | 2.09 ( 1.18 - 3.69 ) | 2.09 ( 6.68 ) | 2.07 ( 1.28 ) | 1.05 ( 0.24 ) |
|  | FRACTURED COCCYX | 9 | 3.89 ( 2.01 - 7.55 ) | 3.89 ( 18.81 ) | 3.81 ( 2.19 ) | 1.93 ( 1 ) |
|  | THORACIC VERTEBRAL FRACTURE | 9 | 2.17 ( 1.12 - 4.19 ) | 2.17 ( 5.59 ) | 2.15 ( 1.24 ) | 1.11 ( 0.18 ) |
|  | SPINAL FUSION FRACTURE | 1 | 33.64 ( 3.76 - 300.97 ) | 33.64 ( 25.33 ) | 27.11 ( 4.33 ) | 4.76 ( 2.4 ) |

**Abbreviations:** ROR: Report Odds Ratio, PRR: Proportional Reporting Ratio, IC: Information Component , EBGM: Empirical Bayesian Geometric Mean
